# Preparation of High-Purity Silicon Carbide Ceramics by Hot Pressing Sintering

**DOI:** 10.3390/nano15110825

**Published:** 2025-05-29

**Authors:** Chang Zou, Yifan Xiao, Lixia Li, Jiagang Chen, Wei Sun, Xingzhong Guo

**Affiliations:** 1 State Key Laboratory of Silicon and Advanced Semiconductor Materials, School of Materials Science and Engineering, Zhejiang University, Hangzhou 310058, China; 0924054@zju.edu.cn (C.Z.); 22426084@zju.edu.cn (Y.X.); 2 Hangzhou Global Scientific and Technological Innovation Center, Zhejiang University, Hangzhou 311200, China; 3 Wuxi Hygood New Technology Co., Ltd., Wuxi 214105, China; lilixia@hy-good.com (L.L.); chenjiagang@hygood.com (J.C.); sunwei83@tsinghua.org.cn (W.S.)

**Keywords:** silicon carbide ceramic, wafer carrier, hot pressing sintering, microstructure, mechanical property, high purity

## Abstract

Wafer carriers, as one of the key components in semiconductor manufacturing processes, have strict purity requirements. In this work, high-strength and high-purity SiC ceramics were fabricated using hot pressing sintering (HPS) combined with particle gradation. After hot pressing sintering with the addition of 15 wt% nano-SiC, a sintering temperature of 2200 °C, and a pressure of 40 MPa, the resultant SiC ceramics demonstrated excellent comprehensive properties with a high purity of 99.967%, a flexural strength of 458.71 MPa, a Vickers hardness of 23.31 GPa, and a fracture toughness of 4.06 MPa·m^1/2^. The high-strength and high-purity SiC ceramics show potential to function as wafer carrier materials in semiconductor manufacturing processes.

## 1. Introduction

Silicon carbide (SiC) ceramic is one of the classic structural ceramics, and exhibits outstanding mechanical properties, superior chemical stability, and exceptional creep resistance, owing to the strong covalent Si-C bonds [1,2,3,4]. Due to their excellent high-temperature properties, SiC ceramics can be applied as wafer carriers, which are essential components in semiconductor manufacturing processes [5,6]. As precision apparatuses in semiconductor fabrication, wafer carriers are required to maintain substantial mechanical strength while simultaneously displaying exceptionally high purity. This is imperative for preventing metallic impurities from adversely impacting semiconductor wafer processing. Consequently, the purity of both quartz and silicon carbide (SiC) wafer carriers should typically exceed 99.9% (3N standard). For more advanced semiconductor manufacturing enterprises, these purity standards are often elevated to 4N (99.99%), 4.5N (99.995%), or even higher.

However, it should be noted that conventional SiC wafer carriers consist of a substrate and high-purity SiC coating; chemical vapor depositing (CVD) is used to apply a high-purity (99.99%) SiC film on the surface of substrates. But CVD SiC composite carriers carry a risk of coating detachment and particle contamination, which cannot simultaneously enable the high purity, high mechanical performance, and long life-time requirements for wafer carriers to be met. To address this critical challenge in the application of SiC ceramic materials within the semiconductor industry, it is necessary to explore an advanced ceramic fabrication method for producing high-purity SiC ceramics with good mechanical properties.

Thus, in this work, we demonstrate the preparation of high-purity SiC ceramics by combining hot pressing sintering (HPS) with nano-SiC powder as an additive. The effects of the nano-SiC powder proportion and sintering temperature on the sintering behavior, microstructure, and mechanical properties of high-purity SiC ceramics are investigated in detail. The resultant SiC ceramics possess high purity and good mechanical properties, and show potential for use as wafer carriers.

## 2. Materials and Methods

Micrometer SiC powders (α-SiC, 2 and 5 μm, >99.999%), nano-SiC powder (β-SiC, 40 nm, >99.9%), polyethylene glycol (PEG), polyvinyl alcohol (PVA), and deionized water were mixed at a specific ratio to form a slurry. The slurry was then ball-milled for 4 h to obtain a slurry with a solid content of 40 wt%. The obtained aqueous slurry was spray-dried to prepare the granulation powder. The composite powder was uniaxially pressed at 100 MPa in a carbon steel die into rectangular specimens, which then were compacted at 250 MPa in a cold isostatic press to obtain a dense green body. The green body was sintered in the HPS furnace under Ar conditions. In the sintering procedure, the furnace was firstly heated up to 400 °C with a heating rate of 6 °C/min, and after insulation for 15 min, was further heated up to the sintering temperature (2100, 2150, or 2200 °C) at a rate of 10 °C/min for 2 h. In addition, before being heated, the green body sample was pre-loaded with a pressure of 30 MPa for 5 min. After that, an initial external pressure of 5 MPa was applied at the beginning of the heating, which was continuously increased as the heating process proceeded, and the external pressure was 40 MPa at the holding stage and maintained until the end of the sintering process. The high-purity SiC ceramic materials were obtained after cooling, demolding, and machining.

The bulk density of SiC ceramic was determined using the Archimedes principle. The conventional water displacement method was used to measure the densities of the SiC ceramic. Phase identification was performed through the X-ray diffraction (XRD, Rigaku D/max-RA X-ray diffractometer, Rigaku, Tokyo, Japan) method using nickel-filtered Cu-Kα radiation in the range of 10–80° at a scanning rate of 2° per minute. The content of metallic impurities in the ceramic samples was determined using Inductively Coupled Plasma Optical Emission Spectrometry (ICP-OES, PerkinElmer ICP 2100, PerkinElmer, Waltham, MA, USA), which allowed for the quantitative determination of the impurity content in the test samples, which was subsequently used to calculate the purity of the high-purity silicon carbide ceramics. The morphology and elemental composition of the SiC powder and sintered samples were examined using scanning electron microscopy (SEM, Thermo Fisher Scientific Scios2 Hivac, Thermo Fisher, Waltham, MA, USA). The microhardness of SiC ceramic materials was measured using an HV-5 Vickers hardness tester with a load of 98 N and a loading time of 15 s. The bending strength and elastic modulus of ceramic test specimens (3 × 4 × 36 mm, 10 samples for each) were measured using a CMT5205 electronic universal testing machine, employing a three-point bending test with a 30 mm span at a crosshead speed of 0.5 mm/min. And the fracture toughness of the material was calculated using the following formula:

$$K_{IC}=\frac{0.129}{3}{\left(\frac{c}{a}\right)}^{-\frac{3}{2}}H\sqrt{a}{\left(\frac{H}{3E}\right)}^{-0.4}\;(\frac{c}{a}>2.5)$$

$$K_{IC}=\frac{0.035}{3}{\left(\frac{l}{a}\right)}^{-\frac{1}{2}}H\sqrt{a}{\left(\frac{H}{3E}\right)}^{-0.4}\;(\frac{c}{a}<2.5)$$

wherein K_IC_ is the fracture toughness of the testing material, H is the hardness of the testing material, E is the elastic modulus of the testing material, l is the indentation crack length, a is the half-length of the indentation diagonal, and c = 1 + a.

## 3. Results and Discussion

**Figure 1:**
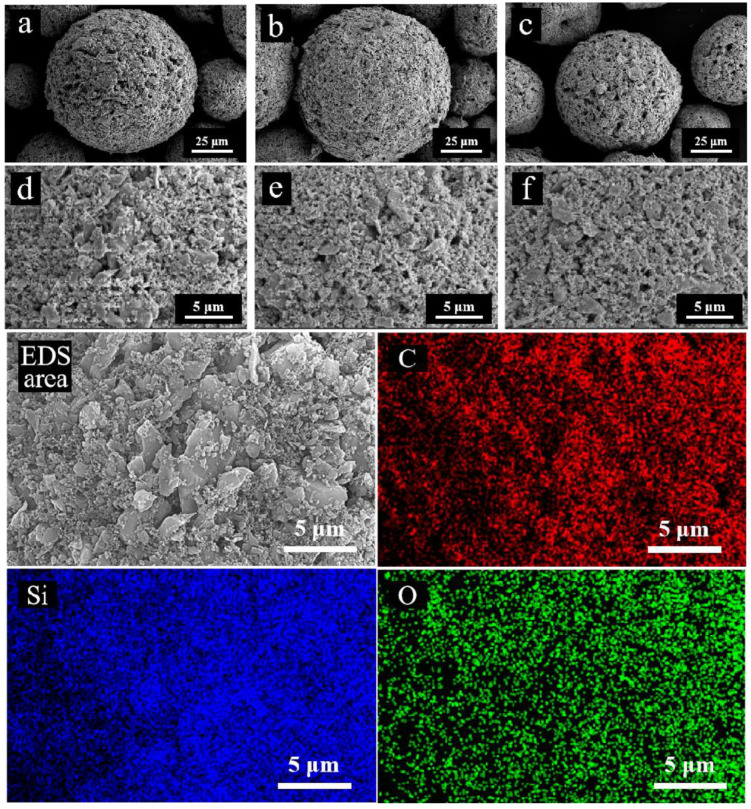
SEM photos of SiC powders with (**a**,**d**) 10 wt% nano-SiC, (**b**,**e**) 15 wt% nano-SiC, and (**c**,**f**) 20 wt% nano-SiC, and (EDS area) EDS photos of SiC powder with 15 wt% nano-SiC.

Figure 1a–f illustrate the morphology of the SiC granulated powders with different nano-SiC contents prepared via ball milling and spray drying. The granulated powder exhibits a particle size ranging from 40 to 80 µm and a regular spherical shape, indicating favorable flowability and high packing density [7,8]. As demonstrated by the EDS photos in Figure 1, the constituent elements Si and C are uniformly distributed throughout the powder, and there are no other metallic impurities. These results confirm the uniform distribution of nano-SiC within the SiC granulated powder, which is beneficial for improving the sintering performance of high-purity SiC ceramics.

**Figure 2:**
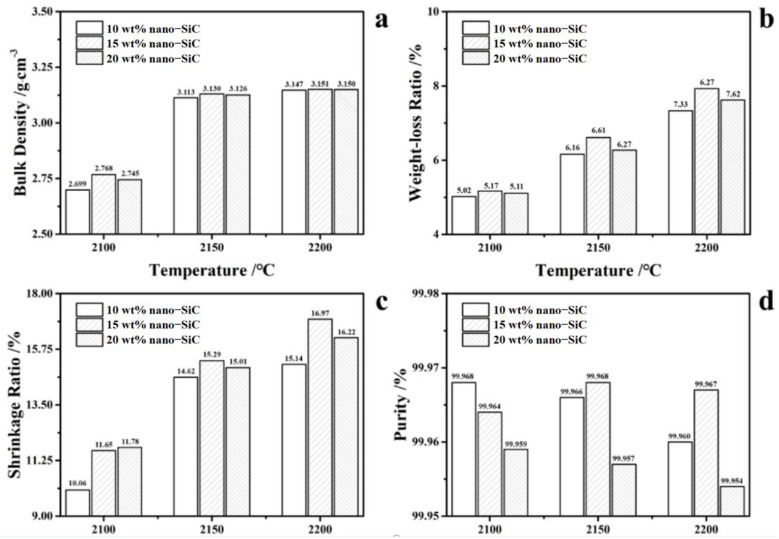

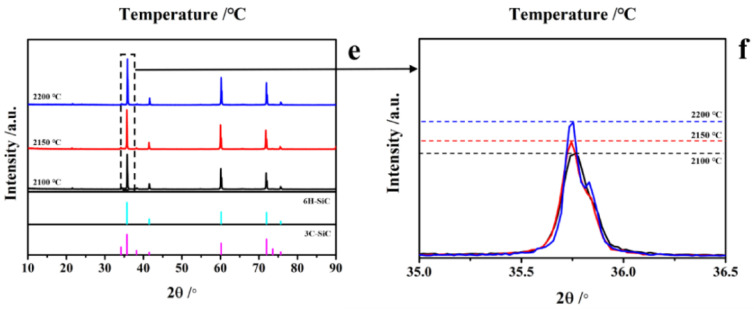
Sintering properties (**a**–**d**) of SiC ceramics with different nano-SiC contents, and XRD patterns (**e**,**f**) of SiC ceramics with 15 wt% nano-SiC sintered at different temperatures.

Figure 2 shows the sintering behavior of the SiC ceramics with different nano-SiC contents after hot pressing sintering at different temperatures. Among the ceramic samples sintered at the same temperatures, the bulk density and shrinkage ratio of the ceramic samples actually show a trend of firstly increasing and then decreasing with the increasing proportion of nano-SiC, while the purity of the ceramic decreased to some extent (Figure 2d). This indicates that the incorporation of nano-SiC improves the sintering densification of the high-purity SiC ceramic, while excessive nano-sized SiC powder hinders the densification of the ceramic. The increased nano-SiC content in ceramic green bodies promotes SiC sintering activity and densification by filling inter-particle voids and enhancing the contact area. However, the excessive addition of nano-SiC (β-phase) leads to overall performance degradation due to the mismatched sintering behavior with the primary α-SiC powder. At the same time, considering that the cost of nano-SiC is higher, it should also be used in moderation from an economic perspective. It is also observed that at the same nano-SiC content, the sintering properties of the samples shows a significant increasing trend with the rise in the temperature, which indicates that the sintering temperature significantly influences the sintering behavior of SiC ceramics. Thus, the SiC ceramics sintered at 2200 °C with 15 wt% nano-SiC addition exhibit the highest density and relative density, which were 3.151 g/cm^3^ and 98.46%, respectively, with a purity of 99.967%. It can be observed from Figure 2e that the primary phase of the high-purity SiC ceramic specimens is 6H-SiC, which is the hexagonal polymorph of α-SiC, and there is a minor presence of 3C-SiC, which is the cubic polymorph of β-SiC. This is because the β-SiC in the raw materials undergoes a gradual phase transformation to α-SiC at temperatures above 1200 °C [9,10]. It should be noted that the transformation is not complete, and a small amount of 3C-SiC remains. The characteristic peaks of 6H-SiC gradually increase with the increase in sintering temperature (Figure 2f), indicating that the phase transformation from β-SiC to α-SiC is more complete at a higher sintering temperature.

**Figure 3:**
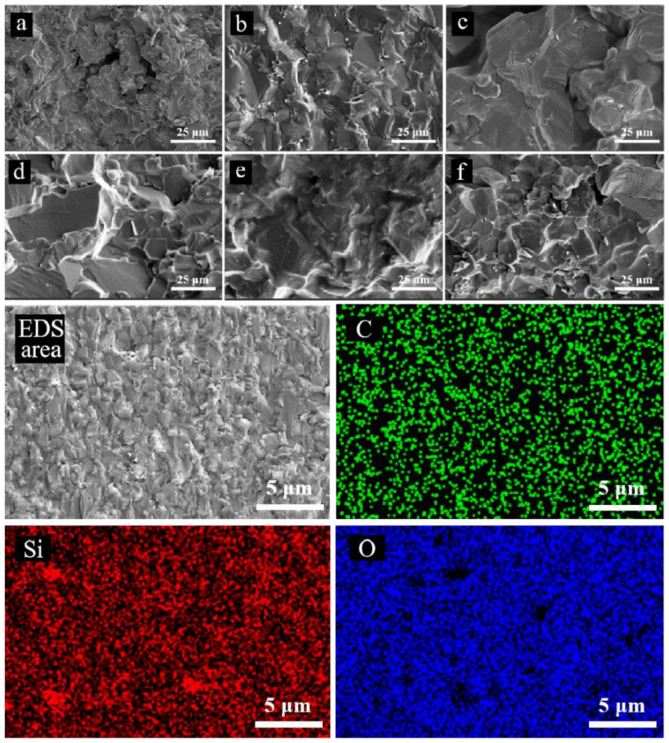
SEM photos of fracture surface of SiC ceramics with 15 wt% nano-SiC after sintering at (**a**) 2100 °C, (**b**) 2150 °C, and (**c**) 2200 °C; SEM photos of SiC ceramics with (**d**) 10 wt% nano-SiC, (**e**) 15 wt% nano-SiC, and (**f**) 20 wt% nano-SiC sintered at 2200 °C; the (EDS area) EDS of SiC ceramics with 15 wt% nano-SiC sintered at 2200 °C.

Figure 3 shows the microstructure of the SiC ceramics with different nano-SiC contents after hot pressing sintering at different temperatures. It is noted that the compactness of the SiC ceramics is enhanced with the increase in the sintering temperature, while the grain size of the SiC ceramics also increases. There exists numerous pores and detached small particles in the sample sintered at 2100 °C, indicating weak densification. During the hot pressing sintering process of the SiC ceramic, mass transfer processes such as contact, diffusion, and flow will occur at the contact surfaces between SiC particles, which leads to gradual bonding and, eventually, the formation of a dense ceramic compact [11]. It is also found that the compactness of the SiC ceramics remains with the increase in the nano-SiC content, while the grain size of the SiC ceramics decreases to some extent. It can be observed from EDS that the element distribution in the fracture surface is relatively uniform after hot pressing sintering.

**Figure 4:**
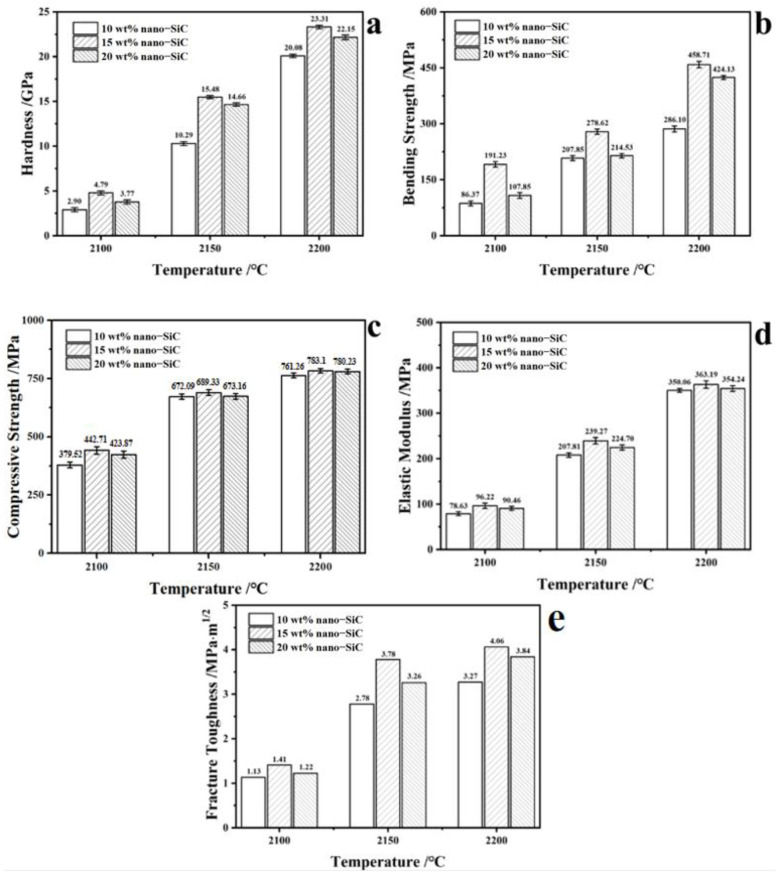
(**a**) Hardness, (**b**) bending strength, (**c**) compressive strength, (**d**) elastic modulus and (**e**) fracture toughness of high-purity SiC ceramics after hot pressing sintering at different sintering temperatures with various nano-SiC contents.

Figure 4 displays the mechanical properties of the SiC ceramics sintered at different sintering temperatures and nano-SiC proportions. The results demonstrate that choosing a high sintering temperature and adding suitable nano-SiC can promote the densification of SiC ceramics and simultaneously refine the grains of SiC ceramics during the sintering process [12,13]. The SiC ceramic sintered at 2200 °C with 15 wt% nano-SiC addition exhibits the highest hardness, bending strength, compressive strength, elastic modulus, and fracture toughness, which were 23.31 GPa, 458.71 MPa, 783.1 MPa, 363.19 MPa, and 4.06 MPa·m^1/2^, respectively. The application of external pressure and the incorporation of nano-powders during hot pressing result in high-purity SiC ceramics with substantially greater densification and superior overall properties than those achieved through recrystallization or pressureless sintering at identical temperatures.

## 4. Conclusions

In summary, high-purity SiC ceramics with excellent sintering performance and mechanical properties have been successfully fabricated via hot pressing sintering with particle gradation of SiC powders. The effects of the nano-SiC proportion and sintering temperature on the sintering behavior, microstructure, and mechanical properties of the SiC ceramics were investigated in detail. The high-purity SiC ceramics prepared at 2200 °C with the addition of 15 wt% nano-SiC under an external pressure of 40 MPa exhibited the highest mechanical properties, achieving a bending strength of 458.71 MPa, a Vickers hardness of 23.31 GPa, and a fracture toughness of 4.06 MPa·m^1/2^, and at the same time, the purity of the SiC ceramics reached 99.967%. The obtained high-purity SiC ceramics hold potential for applications in wafer carriers, which are essential for semiconductor processing.

## Data Availability

The original contributions presented in this study are included in the article. Further inquiries can be directed to the corresponding author.
